# Blood RNA Signatures Enable Accurate Discrimination of Stroke Subtype and Onset Time at Hospital Admission

**DOI:** 10.21203/rs.3.rs-9022364/v1

**Published:** 2026-03-08

**Authors:** Rashi Verma, Andrea Pearson, Zulma Reyes-Benitez, Harriet Blankson, Tallus Haward, Srikant Rangaraju, Alex Hall, Alaina Williams, Nicholas Stanley, Samayah Boynton, Floyd Stern, Tina Toosi, Tiera Bates, Jessica Garcia, Nicholas Liu, Rhidika Zakaria, Caidyn Ellis, Gloria Centeno, Gavin Hurn, Emine Guven, Nirav Bhatt, Roger Simon, I Jordan, Michael Frankel, Robert Meller

**Affiliations:** Morehouse School of Medicine; Morehouse School of Medicine; Morehouse School of Medicine; Morehouse School of Medicine; Morehouse School of Medicine; Yale University; Emory University School of Medicine; Emory University School of Medicine; Emory University School of Medicine; Emory University School of Medicine; Emory University School of Medicine; Emory University School of Medicine; Emory University School of Medicine; Emory University School of Medicine; Emory University School of Medicine; Emory University School of Medicine; Emory University School of Medicine; Emory University School of Medicine; Emory University School of Medicine; Morehouse School of Medicine; University of Pittsburgh School of Medicine; Morehouse School of Medicine; Georgia Institute of Technology; Emory University School of Medicine; Morehouse School of Medicine

## Abstract

Despite advances in thrombolytic therapy (IVT) and mechanical thrombectomy (MT) for acute ischemic stroke, their benefit is strongly time-dependent and contingent on rapid exclusion of intracranial hemorrhage (ICH), where thrombolysis can be fatal. Here we evaluated whether a peripheral blood transcriptome–based machine learning model could rapidly identify hemorrhagic stroke, distinguish ischemic stroke from mimics, and predict eligibility for thrombolysis. Whole-blood samples (n=314) were collected from acute stroke patients admitted at emergency department of Grady Memorial Hospital (Atlanta, GA). Two independent training (n=192) and validation cohorts (n=122) were sequenced, aligned to GRCh38, and quantified with StringTie2. Differentially expressed transcripts were used to train hierarchical machine-learning models (caret, hidden Markov models [HMMs]) to classify hemorrhagic stroke, then distinguish ischemic stroke from stroke mimics, and further predict thrombolysis eligibility (≤3.5 hours from onset) and stroke severity (NIHSS). HMM-based classifiers demonstrated robust performance: a three-transcript panel perfectly discriminated hemorrhagic from non-hemorrhagic stroke (100% accuracy), and a four-transcript panel achieved 97% accuracy with 100% sensitivity and 96% specificity in validation. Ischemic stroke panels accurately distinguished patients from stroke mimics, time-associated transcripts identified individuals within the thrombolysis window, and severity-associated RNA signatures strongly correlated with NIHSS scores. The findings indicate that RNA profiling at admission can rapidly identify stroke subtypes, time of stroke onset, and stroke severity, supporting point-of-care triage and timely thrombolytic therapy.

In the United States nearly 800,000 new and recurrent strokes occur each year, making it the fourth leading cause of death in the US^1^. Stroke is also the leading cause of disability in the US, and stroke-related costs are estimated at $25 billion between 2020-2021, mostly attributed to health care costs^2^. There are substantial geographic disparities in stroke mortality, with higher stroke mortality rates in the southeastern United States (aka the Stroke Belt), which is attributed to challenges in providing timely stroke care^2–5^. Therefore, it is imperative that we improve stroke care in the US.

A rapid stroke subtype diagnosis is necessary for thrombolytic therapy. Intravenous Thrombolytic (IVT) therapy has been proven to be a highly effective therapy for acute ischemic strokes^6–10^, however, this therapy is extremely time-sensitive and exclusion of intracranial hemorrhage is critical prior to safe administration of IVT. A delay in receiving an actionable stroke diagnosis is the primary reason IVT is not administered to a stroke patient. Intracranial hemorrhage must be excluded from the diagnosis using CT imaging prior to IVT administration. The national consensus guidelines for IVT therapy in acute ischemic stroke suggest a therapeutic time window of 4.5h following the onset of stroke symptoms. While certified stroke centers are required to have on-call acute stroke teams and dedicated imaging equipment, smaller and more rural hospitals often lack the resources to ensure timely accurate diagnosis of ischemic stroke^11–14^. Not only do national guidelines require a door to imaging delay of less than 20 min, interpretation of the results by a radiologist of 20-40 min, and a door to needle time of less than 60 min^7^, there is ample evidence that faster treatment leads to less disability and death. A further challenge in rural regions is the fact that many patients reside outside of a 60-90 min travel time to a hospital. The use of mobile stroke units (ambulances fitted with CT imaging and personal trained in IVT administration) can accelerate delivery of IVT, but their cost and other challenges prevent widespread adoption^15^. Together these factors cause diagnostic delay, such that the patient is often outside the IVT therapy window.

The need for rapid stroke subtype determination is also shown by a recent clinical trial reporting rapid anti-hypertensive therapy in the pre-hospital setting (before brain imaging) in patients with hemorrhagic stroke reduced poor outcomes^16^. This underscores the urgent need for alternative approaches to confidently exclude intracranial hemorrhage when CT imaging for patients with acute stroke symptoms is delayed or unavailable, to enable timely therapeutic intervention with IVT.

In the absence of witnessed symptom onset, the ability to determine the onset of a stroke event in patients with aphasia or neglect is also a critical issue for IVT administration. Furthermore, a substantial proportion of strokes occur during sleep. Wake-up strokes comprise approximately 14-30% of ischemic strokes^17^. Such patients are excluded from IVT therapy if the time of stroke onset is unknown or cannot be verified. MRI-based imaging studies show that identifying patients with stroke onset within 4.5h would increase eligibility for IVT administration^18^. However, this level of imaging-based triage is mainly limited to specialized centers. Therefore, developing other approaches to identify patients within the therapeutic window would expand access to IVT and improve acute stroke care.

As an alternative approach to imaging for stroke diagnosis, our research and others have focused on detecting RNA expression changes in blood following a stroke^19–22^. Circulating immune cells act as sentinels responding to pathophysiological conditions in the brain and released “foreign” cell fragments and components in blood^23^. These immune cells react to disease and changing environments by changing their RNA expression^20,24^. RNA expression patterns can encode multiple clinical elements of a stroke, severity of insult, stroke subtype, and prognosis^19,21,22,25,26^. In our previous report we show differences in blood RNA expression following stroke^19^. While these findings are compelling, many such studies have relied on blood samples collected 24h following the onset of stroke symptoms. Here, we prospectively collected blood samples at admission in the emergency room or in a mobile stroke unit to determine whether whole blood transcriptome analysis can distinguish hemorrhagic stroke from ischemic stroke.

## Results

### RNA sequencing,

We performed RNA sequencing on 314 blood samples. On average we created 7.75M reads/sample (set 1: 4.76M, set 2: 7.27M, set3: 11.21M). For our pipeline we removed rRNA associated reads prior to alignment to the human genome. Subsequent StringTie based transcript identification and quantification identified in additional transcripts vs the ensemble model. Novel RNAs that were identified in multiple samples were given the name MSTRG.XXX (XXX denotes the number). A gtf containing genomic coordinates of the MSTRG transcripts is available on request.

### Study Participants

**Fig. 1.**
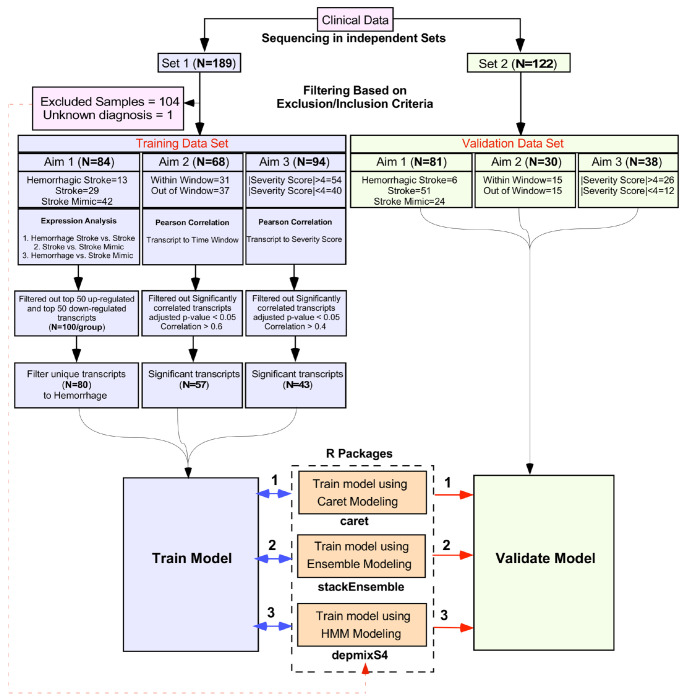
Study design and model development workflow. Clinical samples were sequenced in two independent sets and filtered based on predefined inclusion/exclusion criteria, resulting in a training dataset (Set 1) and an independent validation dataset (Set 2). The training dataset was analyzed across three aims: 1) stroke type classification; 2) Prediction of actionable time of stroke onset; and 3) Prediction of NIHSS stroke severity . Significant transcripts from all aims were combined to train predictive models using multiple approaches, including Caret-based modeling, ensemble modeling (stackEnsemble), and hidden Markov model (HMM)–based modeling (depmixS4). The trained models were then evaluated on the independent validation dataset, stratified by the same aim-specific criteria, to assess model performance and robustness.

RNA sequencing was performed to create two datasets, a training dataset (Set 1:192 samples) and a validation dataset (ComBat-seq corrected Set 2 and 3:122 samples). In the training dataset, 189 had complete clinical data. For stroke type classification, only confirmed hemorrhagic, ischemic, and stroke mimic diagnosis were included. In addition, the following conditions were excluded from training (TIA cases (n=24), patients with unknown diagnoses (n=3), mechanical thrombectomy (n=16), or recurrent/multifocal stroke (n=5). Ischemic stroke patients outside the IVT window with mild deficits (NIHSS <5; n=50) and samples with low read depth (n=7) were also excluded. This yielded 84 high-quality training samples (13 hemorrhagic stroke, 29 ischemic stroke, 42 stroke mimic) (Fig. 1, **Table 1**).

For validation, 108 had complete clinical data. Samples with unknown or ambiguous diagnoses (n = 13), low read depth (n = 12), and malignant conditions (n = 2) were excluded. Unlike the training set, TIA cases, ischemic strokes presenting outside the IVT treatment window, and patients with mild symptoms (NIHSS <5) were retained to evaluate the model’s generalizability to a broader range of clinical presentations. This yielded 81 validation samples (6 hemorrhagic stroke, 51 ischemic stroke, 24 stroke mimic) (Fig. 1, **Table 1**).

### Differential expression profiles between hemorrhagic and ischemic stroke

**Fig. 2.**
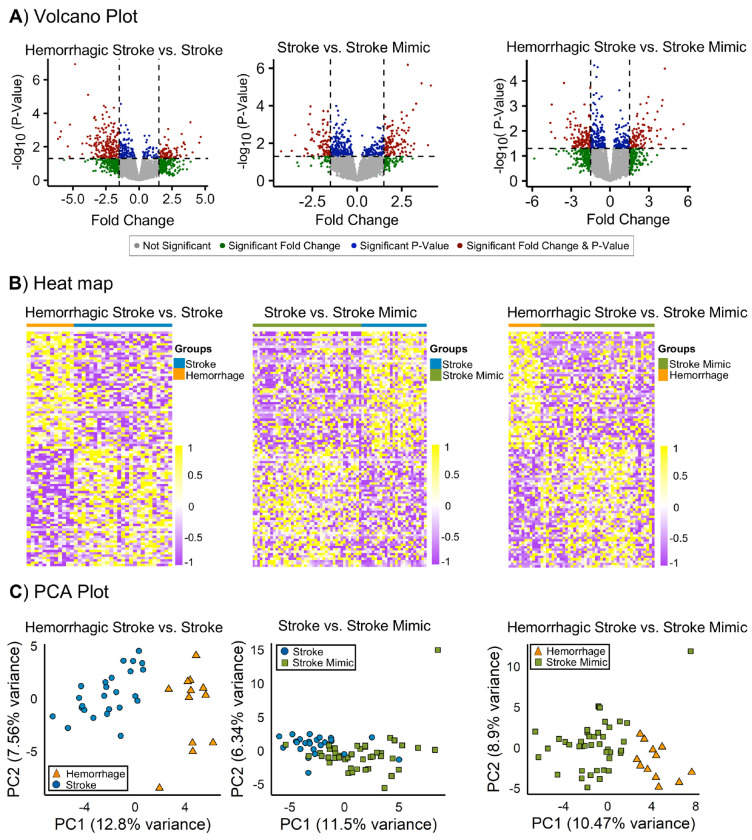
Transcnptomic differences among stroke subtypes. **(A)** Volcano plots illustrating pairwise differential expression analyses between hemorrhagic stroke, ischemic stroke, and stroke mimic groups. Dashed lines indicate fold-change and false discovery rate–adjusted significance thresholds. **(B)** Heat map displaying the top 100 differentially expressed transcripts (50 up-regulated and 50 down-regulated) across stroke subtypes. **(C)** Principal component analysis (PCA) of differentially expressed transcripts expression matrices for each comparison, with the first three principal components accounting for 20.36% (hemorrhagic vs ischemic), 17.84% (ischemic vs mimic), and 19.37% (hemorrhagic vs mimic) of total variance. [Stroke = Ischemic Stroke].

Differential expression analysis was performed on the training dataset for three comparisons: (1) hemorrhagic vs. ischemic stroke, (2) ischemic stroke vs. stroke mimic, and (3) hemorrhagic stroke vs. stroke mimic (Fig. 2). Full matching reduced baseline differences in clinical severity and matched-set weights confirmed balanced covariates without over-representation (**Suppl. Fig 1**). We identified 439 transcripts differentially expressed between hemorrhagic and ischemic stroke, 453 between ischemic stroke and mimics, and 431 between hemorrhagic stroke and mimics (1.5-fold, p<0.05). The overlap and distribution of transcripts are shown in Fig. 2A. A heatmap of the top 100 transcripts showed distinct expression patterns across groups (Fig. 2B), and the subsequent principal component analysis (PCA) further confirmed this separation, clearly distinguishing hemorrhagic stroke from ischemic stroke (20.36% variance) and stroke mimics (19.37%), with only modest separation between ischemic stroke and mimics (17.84%) (Fig. 2C).

**Fig. 3.**
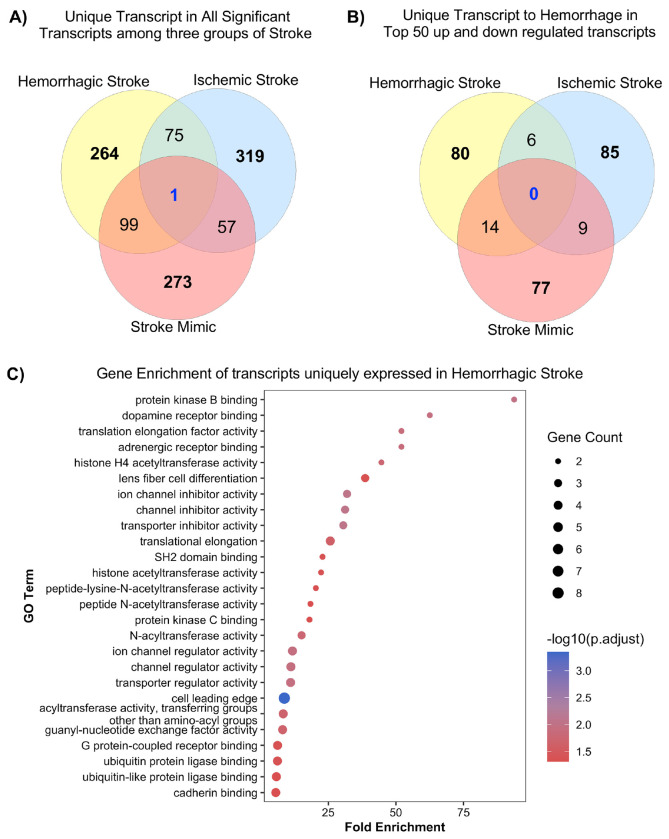
Gene Ontology (GO) analysis of uniquely differentially expressed transcripts in hemorrhagic stroke. **(A)** Venn diagram depicting uniquely differentially expressed transcripts across stroke types using all statistically significant transcripts. **(B)** Venn diagram showing overlap of the top 100 DEGs (50 up-regulated and 50 down-regulated) among stroke types. **(C)** GO enrichment analysis of top DEGs uniquely associated with hemorrhagic stroke.

To characterize condition-specific transcriptional changes, we compared significantly altered transcripts across hemorrhagic stroke, ischemic stroke, and stroke mimics. Venn analysis revealed both shared and distinct signatures (Fig. 3). Among all differentially expressed transcripts, 264 were unique to hemorrhagic stroke, 319 to ischemic stroke, and 273 to stroke mimics, with only one transcript common to all three groups (Fig. 3A). Analysis the top 50 upregulated and top 50 downregulated transcripts for each condition showed limited overlap. Six transcripts were shared between hemorrhagic and ischemic stroke, 14 between hemorrhagic stroke and stroke mimics, and nine between ischemic stroke and stroke mimics, with no transcript common to three groups. Overall, 80 transcripts were unique to hemorrhagic stroke, 85 to ischemic stroke, and 77 to stroke mimics, highlighting distinct molecular signatures associated with each condition (Fig. 3B).

### GO Analysis of Uniquely Expressed Transcripts in Hemorrhagic Stroke

Gene enrichment analysis of transcripts unique to hemorrhagic stroke (n=45/80 protein coding) revealed significant functional enrichment related to protein homeostasis and synaptic regulation. Both BiNGO and clusterProfiler consistently identified metabolic and post-translational modification pathways, with clusterProfiler showing strong fold enrichment and BiNGO illustrating hierarchical relationships among terms. Notably, several acetyltransferase activities were significantly enriched, including histone acetyltransferase activity, peptide-lysine-N-acetyltransferase activity, and N-acetyltransferase activity, suggesting epigenetic involvement. Additional enriched categories included SH2 domain activity, protein kinase C binding, ion channel and transporter regulation, translation elongation, and GTPase and GPCR-related signaling (Fig. 3C). These results highlight key molecular processes, including post-translational modification, signal transduction, and membrane transport, that may contribute to hemorrhagic stroke pathophysiology.

### Prediction of Hemorrhagic Stroke Diagnosis using RNA Signatures

**Fig. 4.**
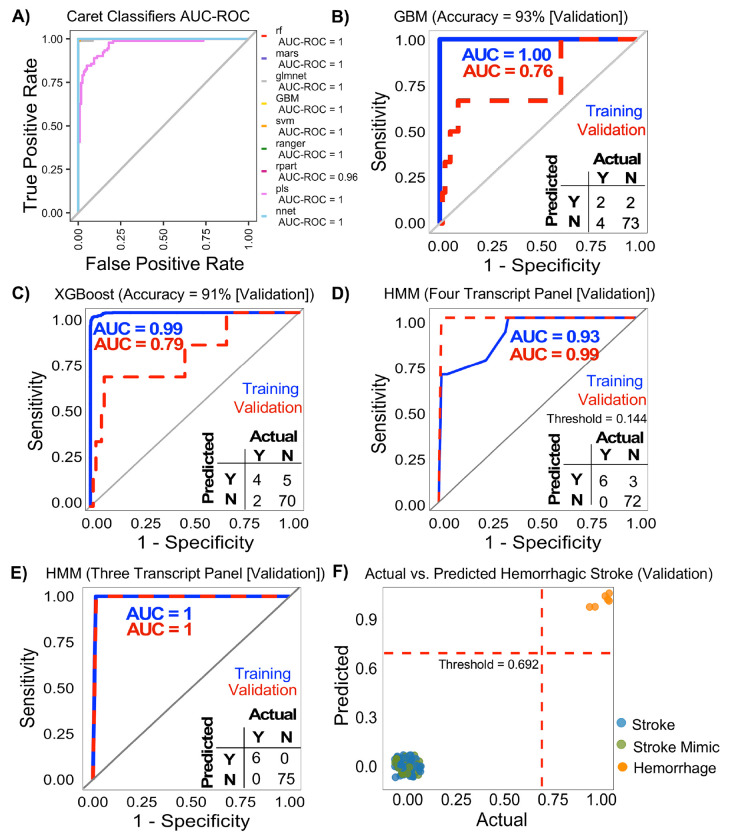
Prediction of Hemorrhagic Stroke Diagnosis. **(A)** ROC curves for multiple models showing high performance (AUC, 0.39–1.00). ROC curve for the **(B)** GBM model and **(C)** XGBoost using soft voting (AUC = 0.76) and confusion matrix for the best validation model. (**D**) Validation performance of the four-transcript panel HMM model (ROC-AUC = 0.99) and (**E–F**) three-transcript panel HMM model (ROC-AUC = 1), demonstrating 100% accuracy for hemorrhagic stroke diagnosis.

Transcript expression values unique to hemorrhagic stroke (n=80) effectively distinguished hemorrhagic stroke from ischemic stroke and stroke mimics. Application of SMOTE improved minority class representation and increased sensitivity (from 17% to 67%) for hemorrhagic stroke detection (**Table 2**). Repeated cross-validation (10 folds × 10 repeats) on the training set showed strong predictive performance across several models with average AUC values ranging from 0.96 to 1.00 (Fig. 4A). As expected, performance declined during validation testing. The RF, RANGER, and rSVM achieved high overall accuracy (92%) but failed to correctly identify hemorrhagic stroke cases (0% sensitivity). In contrast, GBM provided the most balanced performance achieving 93% accuracy, 97% specificity, 33% sensitivity, and an AUC of 0.76 (Fig. 4B, **Table 2**). XGBoost also performed well with 91% accuracy with improved sensitivity to 67% (Fig. 4C). Recursive feature elimination (RFE) produced a reduction in AUC, but similar accuracy for all models tested (**Suppl. Fig. 2**).

Evaluation of individual HMMs identified eight transcripts with strong discriminative performance in the training dataset. All possible multi-transcript combinations derived from these eight transcripts were subsequently assessed. A four-transcript panel (**Table 3**) showed excellent validation performance with an ROC-AUC of 0.99 and 100% sensitivity, misclassifying three non-stroke samples (Fig. 4D). Notably, the HMM model based on a three-transcript panel achieved 100% accuracy in distinguishing hemorrhagic stroke from ischemic stroke and stroke mimics, demonstrating strong generalizability and potential clinical utility (Fig. 4E–F). Complete lists of performance-equivalent panels are provided in the **Suppl. Data**.

### Prediction of an Ischemic Stroke Diagnosis using an HMM model

**Fig. 5.**
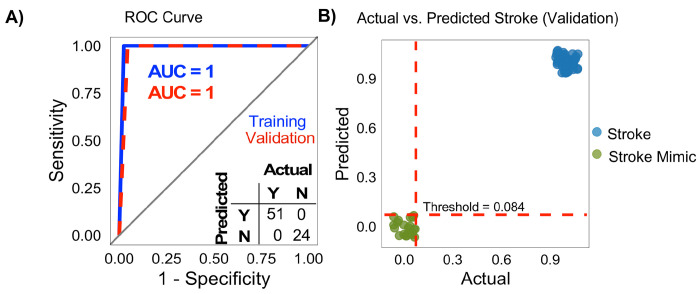
Prediction of Ischemic Stroke Diagnosis. HMM-based classifier to differentiate ischemic stroke from stroke mimics. **(A-B)** ROC curve (ADC = 1.00) and confusion matrix for the best-performing HMM on the validation dataset, demonstrating 100% accuracy (51 true positives, 24 true negatives, 0 false positives, 0 false negatives).

We next developed an HMM-based classifier to differentiate ischemic stroke from stroke mimics. Transcript (85) expression profiles unique to ischemic stroke were evaluated in both the training (n = 72) and validation (n = 78) datasets and retained (Fig. 3B). Individual HMM screening identified 25 transcripts with strong discriminative ability in the training cohort. All possible combinations derived from these 25 transcripts were subsequently assessed. Total seven four-transcript combinations achieved perfect classification performance on both training and validation datasets (**Suppl. Data**). These combinations demonstrated substantial overlap in constituent transcripts, indicating redundancy in the discriminative signal rather than reliance on a single optimal panel. Representative panel was selected using predefined criteria (see method, **Table 3**). Models built on these panels achieved AUC=1.0 in both the training and validation cohorts (Fig. 5A). Threshold optimization identified 0.084 as the optimal cutoff, yielding 100% classification accuracy for distinguishing ischemic stroke from stroke mimics in the validation dataset (Fig. 5B). Complete lists of performance-equivalent panels are provided in the **Suppl. Data**.

### Performance of Model on excluded samples

To further evaluate model generalizability, samples excluded from the initial training datasets were tested separately using the above HMM models. Total 105 samples were initially set aside, of which one sample lacked a final diagnosis and was removed. All 104 samples (99 ischemic, 5 stroke mimics and no hemorrhagic stroke) were further analyzed as independent dataset. For hemorrhagic vs. non-hemorrhagic classification, model correctly classified all samples as non-hemorrhagic, yielding 100% accuracy and specificity. For ischemic stroke versus stroke mimic classification, model achieved perfect classification performance, confirming robust generalization beyond the training and validation datasets (**Suppl. Data**).

### Prediction of actionable time of stroke onset

**Fig 6.**
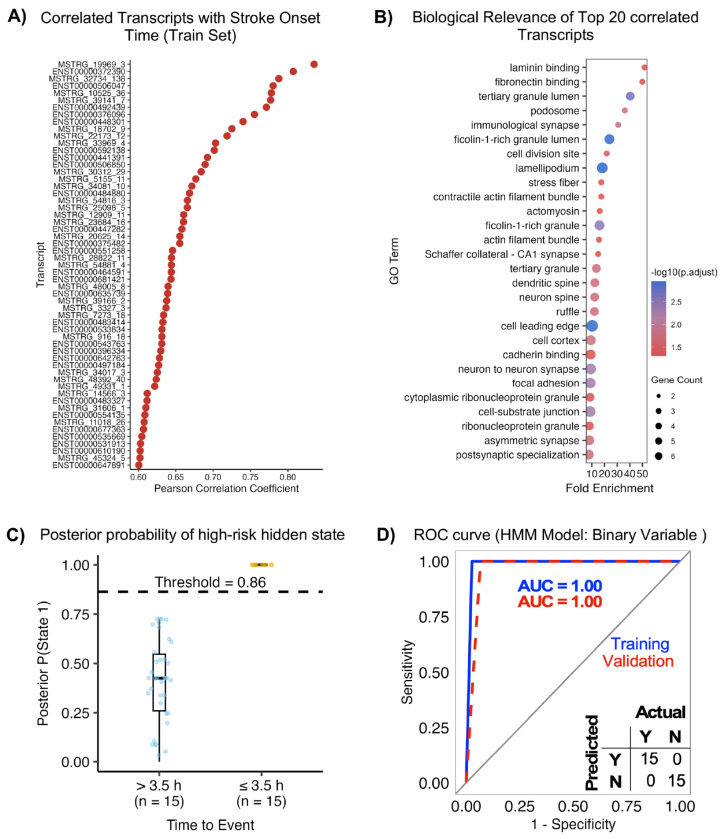
Prediction of actionable time of stroke onset. **(A)** Time-associated transcripts consistently significant in training and validation cohorts (n = 49) showing increasing expression across the post-stroke time window and discriminating patients within (≤3.5 h) versus beyond (>3.5 h) the intravenous tPA therapeutic window. **(B)** Gene Ontology and pathway enrichment analysis of time-associated transcripts highlighting neuronal, cytoskeletal, developmental remodeling, and immune-response processes. **(C)** Posterior probability of high-risk hidden state stratified by time to event. Points represent samples and boxplots summarize distribution. (**D**) Hidden Markov model (HMM)–based classification performance of optimal multi-transcript panels, showing high accuracy across training and validation cohorts.

The time window for thrombolysis is 4.5h post symptom onset. The target door to IVT administration time is 60 min, hence we determined whether patients with a stroke onset within 3.5 h of admission could be identified. We excluded patients missing last known well, recurrent stroke and low-depth samples, to yield 68 acute ischemic stroke RNA samples for HMM modeling in the training dataset and 30 for validation dataset. Pearson correlation analysis identified 140 transcripts whose expression levels were strongly associated with time since stroke onset (|r| ≥ 0.6, FDR < 0.05), with several transcripts demonstrating a monotonic increase (r = 0.60–0.85) across the post-stroke time window (Fig. 6A). These time-associated transcripts were enriched for biological processes and cellular components related to cytoskeletal remodeling, cell adhesion, immune activation, and synaptic organization, including terms such as cell leading edge, lamellipodium, focal adhesion, actomyosin complexes, immunological synapse, and postsynaptic specialization (Fig. 6B), supporting a coordinated temporal response to ischemic injury.

HMM-based classification identified 35 of 57 time-associated transcripts with perfect individual discrimination, which were advanced for multi-transcript modeling. Exhaustive evaluation of transcript combinations yielded 84 optimal panels with consistent performance across datasets. A representative panel was selected using predefined criteria (see Methods, **Table 3**). Key transcripts, particularly ENST00000531913 and ENST00000677363, were recurrently selected alongside novel MSTRG transcripts, underscoring their central role in time-based discrimination (**Suppl. Data**). These panels accurately classified patients within (≤3.5 h) versus beyond (>3.5 h) the intravenous thrombolysis window, demonstrating clinical relevance. Posterior state probability analysis showed clear separation between groups, with optimal performance at K = 2 hidden states (Fig. 6C). Receiver operating characteristic analysis demonstrated perfect discrimination in training and validation cohorts (AUC = 1.00; 100% sensitivity and specificity; Fig. 6D). Strong concordance between predicted and true labels supports the robustness of the HMM-based temporal classification approach.

### Prediction of NIHSS stroke severity rating.

**Fig. 7.**
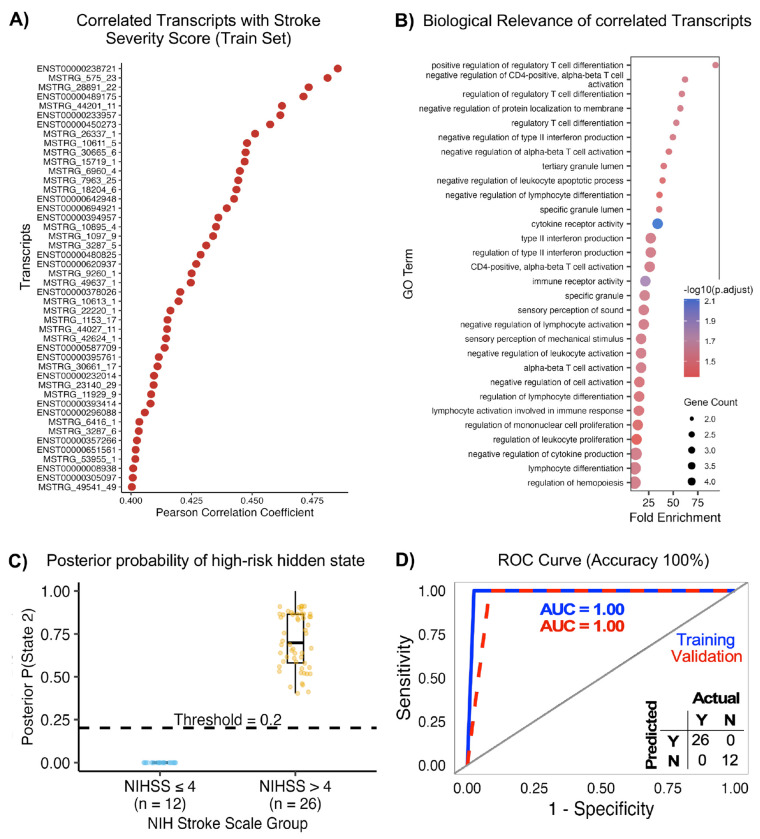
Prediction of NIHSS stroke severity rating. **(A)** Pearson correlation identified 77 transcripts associated with admission NIHSS scores (|r| > 0.6, FDR < 0.05), of which 43 were consistently significant in training and validation cohorts. **(B)** Functional enrichment of correlated transcripts showed over-representation of immune-regulatory and inflammatory pathways, including lymphocyte and T-cell proliferation, cytokine signaling, leukocyte adhesion, coagulation, and ion transport. **(C)** Posterior predicted probabilities showed a clear threshold effect across NIHSS, separating low- from high-severity ranges. **(D)** HMM screening identified 26 transcripts with perfect single-transcript discrimination; three multi-transcript panels achieved 97–99% accuracy in training and 100% accuracy in validation.

Finally for severity-associated profiling, analysis was restricted to AIS cases (n = 105 of 189 total samples). Recurrent or multifocal strokes, which could confound severity-linked transcriptional patterns, were excluded, along with cases missing NIHSS scores and low-depth samples. This yielded 94 high-quality samples for training and 38 for validation. Pearson correlation analysis identified 83 transcripts moderately associated with admission NIHSS scores (|r| > 0.6, FDR < 0.05) (Fig. 7A). Of these, 46 transcripts were consistently significant in both training and validation cohorts (r = 0.40–0.48), Functional enrichment analysis of the top 20 transcripts revealed significant over-representation of immune-regulatory and inflammatory pathways, including negative regulation of T-cell proliferation, negative regulation of lymphocyte proliferation, negative regulation of mono-nuclear cell proliferation and regulation of leukocyte cell-cell adhesion (Fig. 7B). Additionally, enrichment of TNF superfamily cytokine production, inflammatory response to wounding, cytokine regulation, and CD4^+^ T-cell activation indicated amplified immune dysregulation with increasing stroke severity. Pathways related to homeostasis, and ion transmembrane transport were also enriched, reflecting vascular stress and systemic physiological disruption associated with more severe ischemic stroke (Fig. 7B).

HMM modeling of severity-associated transcripts identified 28 of 46 transcripts with perfect discriminative performance in a single-transcript screening, which were advanced for multi-transcript panel construction. Exhaustive testing of possible combinations identified 43 panels with the most stable performance (**Suppl. Data**). A representative panel was selected using predefined criteria (see Methods, **Table 3**). Posterior state probability analysis demonstrated clear separation between severity groups, with samples classified as NIHSS >4 showing high posterior probability for the severe state, while NIHSS ≤4 samples clustered near zero. A probability threshold of 0.2 achieved complete separation between groups (Fig. 7C). Receiver operating characteristic analysis showed perfect discrimination in both training and validation cohorts (AUC = 1.00), with 100% sensitivity and specificity (Fig. 7D). Collectively, these results demonstrate robust and accurate HMM-based prediction of NIHSS stroke severity.

## Discussion

In this study of patients presenting with possible acute stroke symptoms, RNA profiles obtained from admission blood samples combined with AI/ML modelling accurately distinguished amongst hemorrhagic stroke, ischemic stroke, and stroke mimics. A three-transcript HMM classifier identified hemorrhagic stroke with 100% accuracy, and a four-transcript panel achieved 97% accuracy (100% sensitivity, 96% specificity) in validation. Separate panels perfectly differentiated ischemic stroke from stroke mimics. Time-dependent changes in transcriptomic profiles following ischemic stroke enabled the identification of patients within the thrombolysis window with 100% validation accuracy. Further, RNA signatures reflected stroke severity, with greater immune and inflammatory activation in more severe cases. Together, these findings support admission blood RNA profiling as a rapid, clinically actionable tool for acute stroke management.

Our work extends prior studies showing the utility of blood RNA signatures for stroke subtyping^20–22^. These studies primarily analyzed samples collected 3–24h post-stroke and focused on subtype or cryptogenic stroke classification. Here, we analyzed blood collected at initial clinical presentation, including mobile stroke unit samples obtained at the earliest time after symptom onset. A major strength of our approach is the use of a StringTie-based de-novo transcript discovery pipeline, enabling detection of novel RNA species beyond current Ensemble annotations. Consistently detected transcripts were cataloged as MSTRG features, many of which showed strong discriminatory power across stroke subtype, time-window, and severity. Although most lacked functional annotation and were excluded from Gene Ontology analyses, their reproducible expression patterns and predictive value suggest roles as uncharacterized regulatory RNAs, alternative isoforms, or rapidly inducible transcripts involved in acute brain injury and systemic immune responses. With further validation, transcript-based assays could complement neuroimaging and clinical assessment to improve diagnostic precision.

Previous studies of blood-based biomarkers for acute stroke diagnosis have shown limited accuracy and clinical utility^27^. Indeed, many protein biomarkers reflect nonspecific brain injury rather than stroke etiology; for example, S100β is elevated in multiple neurological conditions^28^. While multi-marker protein panels modestly improve performance, they remain insufficient for routine use^29^. GFAP has shown promise for identifying hemorrhagic stroke, particularly in severe cases, but reported accuracy varies widely due to differences in assay methods, sampling times, and cutoff thresholds^30–32^. GFAP levels are also influenced by other brain pathologies, and most studies rely on ROC analyses without validating fixed decision thresholds in independent cohorts^33–35^. Importantly, few studies have established and prospectively validated predefined diagnostic cutoffs^36,37^.

To date, no existing biomarker can reliably estimate time since stroke onset, a key determinant of eligibility for reperfusion therapies. This limitation is especially relevant for wake-up strokes, which account for approximately 25% of ischemic stroke cases and present with unknown symptom onset, frequently excluding patients from acute treatment. To address this gap, we identified temporal RNA expression patterns following stroke and trained predictive models to estimate time since onset.

Transcriptomic analyses also provided biological insight into stroke subtype, progression, and severity. Hemorrhagic stroke signatures were enriched for pathways related to protein homeostasis, post-translational modification, and synaptic regulation, whereas ischemic stroke signatures were dominated by immune and inflammatory pathways, cytoskeletal remodeling, and neuronal structural processes. Time-dependent transcripts were associated with neuronal development, glial migration, and interferon signaling, while severity-associated transcripts were enriched for cytokine-mediated immune pathways, linking greater clinical severity to systemic immune dysregulation.

Using a training–validation framework, we showed that transcriptomic machine learning models achieve high accuracy across multiple clinically relevant tasks. Transcripts quantified in training set (novel and known transcripts) were independently quantified in validation dataset. Batch effect didn’t perform between training and validation dataset to prevent data leakage. While, caret-based models performed well (83% accuracy), Hidden Markov Models showed even more impressive performance. Compact multi-transcript panels derived from HMM screening achieved perfect or near-perfect discrimination of stroke subtype (one error over 300 samples), time since onset relative to the thrombolysis window, and NIHSS-based severity. The probabilistic structure of HMMs enhances robustness to biological variability and supports modeling of latent disease states. Furthermore, the high performance of the HMM model in the held-out samples (set 1/ training) supports generalizability within this dataset and underscores the translational potential of small, targeted RNA panels.

Several limitations should be acknowledged. Current RNA sequencing workflows are too slow for routine emergency department decision-making, particularly for thrombolysis. At present, the full processing and analysis of RNA-seq data can take several hours to days, which limits immediate clinical applicability. However, ongoing advances in targeted RNA assays and rapid sequencing technologies may soon enable clinically feasible and rapid turnaround times. If successfully translated, such assays could support acute stroke triage and earlier treatment, particularly in patients with unclear symptom onset or limited access to advanced neuroimaging. Finally, RNA biomarkers capable of estimating stroke onset and infarct burden may in future complement or reduce reliance on MRI or CT perfusion for patient selection in extended thrombolysis, however, validation across more diverse stroke populations is still needed to enhance generalizability.

## Materials and Methods

### IRB and ethical approval

This study was approved by the Institutional Review Board (IRB) at Morehouse School of Medicine (ID:1007918). Research was overseen by the Research Oversight Committee (ROC) of Grady Memorial Hospital, Atlanta, GA. Only participants who signed the informed consent were enrolled in the study. Samples were de-identified prior to analysis.

### Patient Inclusion/Exclusion Criteria

Patients aged ≥18 years old presenting to the emergency department with acute stroke symptoms were enrolled after providing informed consent. Clinical data was prospectively collected at admission included demographics, anthropometric measurements, vital signs, medication history, and receipt of reperfusion therapy (IVT or MT). All clinical variables were verified by board-certified vascular neurologists through structured assessment and medical record review. Stroke subtype was confirmed through non-contrast CT, and the time of symptom onset or last known well was recorded. Stroke severity was assessed at admission and discharge using National Institutes of Health Stroke Scale (NIHSS). Patients were excluded if they had a history of cancer (except basal cell carcinoma) or prior stem cell transplant, hemorrhagic arteriovenous malformation, malignant hypertension with acute non-CNS organ damage, septic cerebral embolus, acute myocardial infarction, recent bleeding disorders, anticoagulation with INR >3, sepsis or DIC, extreme blood glucose (<30 or >400 mg/dL), creatinine >4 mg/dL, hematocrit <25%, oxygen saturation <90% on room air, chronic dialysis, current neurological or psychiatric disorders affecting evaluation, or recent participation in investigational drug, gene, or cellular therapies.

### Blood Sampling and Data Collection

Venous blood samples were collected from acute stroke patients at admission to Grady Memorial Hospital, or in a mobile stroke unit, as part of routine stroke alert laboratory draws before any treatment. RNA samples (3 ml) were collected in PAXgene tubes, and DNA samples (4 ml) were collected in EDTA tubes. The timing of each blood draw and its relation to IVT administration were documented. After the patient consented, each sample was assigned a unique study identifier. Samples and isolated RNA were stored at −80°C until analysis.

### RNA Sequencing and Alignment

RNA was extracted and eluted in 15 μl and 500 ng was used to prepare barcoded whole-transcriptome libraries with the Ion Total RNA-Seq Kit_v2 (Life Technologies. Thermo Fisher Scientific Inc, CA, USA). Libraries were quantified using the Agilent High Sensitivity DNA Kit, templated on Ion Chef (Thermo Fisher Scientific Inc, MA, USA), and sequenced on the Ion Torrent platform (20–40 million reads/sample) (Thermo Fisher Scientific Inc, CA, USA). Ribosomal RNA reads were removed bioinformatically. Remaining data were converted to FASTQ, trimmed with TrimGalore (v0.6.4), and aligned to GRCh38 using STAR (v2.7.3a) and Bowtie2 (v2.3.5.1)^38,39^. BAM files were assembled and annotated with StringTie2 (v2.2.1)^40^. Counts were combined into a single count matrix file for further investigation (available on request) and transferred to the R package (v4.3-4.5) for further analysis.

### Datasets

Blood samples were analyzed from three independent sets of patients (sample size = 192, 63, and 59). The first set was used to train our model, and the other two sets were combined to test the models. Potential batch effects across datasets two and three were assessed and corrected using ComBat-Seq^41^. All samples were assessed for quality and completeness of associated clinical and medical data, those with missing data were excluded. Only patients with a clear diagnosis of hemorrhagic stroke, ischemic stroke (either IVT treated or time window <3.5-4.5h and NIHSS >5), or stroke mimic were used for training. Patients whose diagnosis were uncertain, had very mild or resolving symptoms, or those with treatments that could change transcript activity in ways unrelated to stroke were excluded from training. In contrast, milder stroke cases and patients with transient ischemic attack (TIA) were retained in the validation dataset.

### Transcriptome Profiling and Feature Selection

RNA transcripts were subjected to feature selection. For stroke type classification (categorical outcome), differential expression analysis was performed using limma (v3.58.1) and edgeR (v3.42.4) on the training dataset with contrasts specified for three pairwise group comparisons: (i) hemorrhagic stroke versus ischemic stroke, (ii) ischemic stroke versus stroke mimic, and (iii) hemorrhagic stroke versus stroke mimic^42,43^. Full matching was performed to minimize confounding between groups using Mahalanobis distance. Transcripts were filtered to remove lowly expressed transcripts, then normalized using TMM and transformed with voom to estimate mean–variance relationships. A linear modeling framework was then applied with a design matrix that included diagnosis while adjusting for age and sex. Transcripts with p-value <0.05 and fold-change ≥1.5 were retained. The top 50 upregulated and top 50 downregulated transcripts were selected for comparison. Duplicate transcripts across comparisons were removed to generate a set of transcripts unique to each condition.

For time window and stroke severity studies, correlations (Pearson’s) were computed between transcript expression and either absolute time since stroke onset or admission NIHSS scores. Transcripts showing significant correlations (FDR <0.05) in the training dataset were retained for downstream analysis (**Script available on GitHub**).

### Gene Ontology (GO) Analysis

Gene Ontology enrichment analysis was performed separately on the transcripts retained from each classifier list (stroke types, time window, severity outcome) using clusterProfiler^44^. The MSTRG genes were excluded from GO analysis. Adjusted p-values were calculated with the Benjamini-Hochberg method to control the false discovery rate (FDR), with significance set at p-value <0.05. Enriched GO terms were visualized using enrichplot and ggplot2 (v3.5.1).

### Modeling and Classifier Development

For stroke type classification, hierarchical pairwise comparisons were performed (1. hemorrhagic stroke was distinguished from ischemic stroke and stroke mimics. 2. Ischemic stroke was compared against stroke mimics). For IVT eligibility, patients were categorized as within (≤3.5 h) or beyond the therapeutic window based on established guidelines^7^ [FDA IVT guideline >/ 4.5h - 60min (AHA door to needle target time) = 3.5h]. For severity-associated profiling, patients were categorized as minor (NIHSS <5) versus more severe stroke (NIHSS >5)^45^.

Selected transcripts for all three tasks were evaluated independently using a combination of machine learning and probabilistic approaches. For machine learning, individual classifiers were implemented using the caret package (v6.0-94), including Random Forest (RF), advance RF (RANGER), Elastic Net (GLM-NET), Multivariate Adaptive Regression Splines (MARS), Partial Least Squares (PLS), Neural Network (NNET), Recursive Partitioning (rPART), Gradient Boosted machine (GBM), and Support Vector Machine (rSVM)^46^. Imbalanced groups, such as hemorrhagic stroke, were balanced using Synthetic Minority Over-sampling Technique (SMOTE)^47^. Additionally, Recursive Feature Elimination (RFE) was performed to assess performance using only the most important features^48^.

Hidden Markov Models (HMMs) were implemented using the depmixS4 package (v1.5-0), with model complexity selected by Bayesian Information Criterion (BIC). Individual transcripts were first screened for discriminatory ability, with perfect separation defined as 100% classification accuracy. Multi-transcript panels were constructed from transcripts showing perfect or near-perfect separation in the training set. Biomarker panel size was constrained a priori to a clinically feasible range of three to five transcripts and evaluated across all tasks^49–51^. Panel selection prioritized consistent performance, threshold stability, and interpretability rather than task-specific peak accuracy. Four-transcript panels served as the primary models to maintain consistent complexity across tasks, while performance-equivalent transcript panels were examined in sensitivity analyses. When multiple panels met all criteria, one representative panel was selected deterministically to ensure reproducible reporting (**Suppl. Data**).

Classification predictions were generated for both training and independent validation datasets, and model performance was assessed using accuracy, sensitivity, specificity, Youden’s J statistic and AUC-ROC (**script available on GitHub**). Model generalizability was further tested on samples excluded from the training dataset, confirming robustness.

## Supplementary Material

This is a list of supplementary files associated with this preprint. Click to download.

5SupplDataRVRM.docx

SUPPLEMENTARYFIGURESlegends.docx

4SupplFigRVRM.pdf

3Tables.docx

Tables are available in the Supplementary Files section.

## Data Availability

R and Linux based codes for the project are available on the github page (https://github.com/Vermarashi/Stroke.git; https://github.com/rob-meller/). Genomic data will be available in dbGAP (phs002285.v1.p1). The PI can also be contacted to request data and codes.
